# Two-component surface replacement implants compared with perichondrium transplantation for restoration of Metacarpophalangeal and proximal Interphalangeal joints: a retrospective cohort study with a mean follow-up time of 6 respectively 26 years

**DOI:** 10.1186/s12891-020-03687-3

**Published:** 2020-10-07

**Authors:** Daniel Muder, Nils P. Hailer, Torbjörn Vedung

**Affiliations:** 1 Department of Surgical Sciences/Orthopedics & Hand Surgery, Uppsala University, Entrance 70, 751 85 Uppsala, Sweden; 2 Department of Orthopedics, Falu Lasarett, Lasarettsvägen 10, 791 82 Falun, Sweden; 3 Elisabeth Hospital, Aleris Healthcare AB, Geijersgatan 20, 752 26 Uppsala, Sweden

**Keywords:** Articular cartilage, Perichondrium, Transplantation, Joint replacement, Reconstruction

## Abstract

**Background:**

The aim of our study was to compare the long-term outcome after perichondrium transplantation and two-component surface replacement (SR) implants to the metacarpophalangeal (MCP) and the proximal interphalangeal (PIP) joints.

**Methods:**

We evaluated 163 joints in 124 patients, divided into 138 SR implants in 102 patients and 25 perichondrium transplantations in 22 patients. Our primary outcome was any revision surgery of the index joint.

**Results:**

The median follow-up time was 6 years (0–21) for the SR implants and 26 years (1–37) for the perichondrium transplants. Median age at index surgery was 64 years (24–82) for SR implants and 45 years (18–61) for perichondium transplants. MCP joint survival was slightly better in the perichondrium group (86.7%; 95% confidence interval [CI]: 69.4–100.0) than in the SR implant group (75%; CI 53.8–96.1), but not statistically significantly so (*p* = 0.4). PIP joint survival was also slightly better in the perichondrium group (80%; CI 55–100) than in the SR implant group (74.7%; CI 66.6–82.7), but below the threshold of statistical significance (*p* = 0.8).

**Conclusion:**

In conclusion, resurfacing of finger joints using transplanted perichondrium is a technique worth considering since the method has low revision rates in the medium term and compares favorable to SR implants.

**Level of evidence:**

III (Therapeutic).

## Background

Articular cartilage is a highly specialized tissue with limited capacity to repair itself. Clinicians and researchers have struggled with this limitation and the problem it causes for centuries [1]. Different surgical methods aiming towards cartilage repair or regeneration have been introduced, e.g. microfracturing methods, mosaicplasty, osteochondral allografting, autologous chondrocyte implantation, periosteal transplantation and perichondrial transplantation [2–5]. However, not all techniques are applicable when the entire joint has to be resurfaced, especially not when it comes to small joints. In parallel, continuous improvement and refinement have made joint implants increasingly more popular during the last decades, also in small joints. Reconstruction of the metacarpophalangeal (MCP) and the proximal interphalangeal (PIP) joints in patients with osteoarthritis or rheumatoid arthritis is challenging, especially in young individuals with a long life expectancy and high physical demands. Insertion of two-component surface replacement (SR) implants is currently a common method to reconstruct osteoarthritic finger joints in the non-rheumatoid patient. A variety of implants are available on the market, and although short-term results may be satisfactory, the long-term outcome after SR implants is sometimes disappointing. A recent meta-analysis indicates that post-operative complications occur in as many as 28% of patients after PIP replacement surgery, with loosening being the most common reason for revision [6]. Variable results and reoperation rates as high as 58% have been reported after SR implants to the PIP join [7].

In today’s perspective, surface replacement of osteoarthritic finger joints with perichondrium harvested from the rib may seem old-fashioned, but it is described as an alternative reconstructive method [8–10]. The surgical technique of perichondrial transplantation was introduced by Skoog et al. in the 1970’s [11], and it was a relatively widespread method during the following decades [12–14]*.* However, partly due to high revision rates in both the MCP- (*n* = 16, revision 19%) and PIP-joints (*n* = 20, revision 30%) reported after minimum follow-up of 3 years by Seradge et al. [13] and partly due to improved SR implants use of this technique subsequently declined. The need for a second surgical site to harvest the graft may also in part explain the limited use of this method [10]*.* Most reports on joint reconstructions in the hand with perichondrium are historical and consist of limited case series on few patients (*n* = 17—26) mostly reporting short-term results [12, 13, 15]. Recently, long-term results after perichondrial transplantation to finger joints were reported [16]. The follow-up time was very long (mean 37 years) and the results were gratifying in most cases. However, comparisons with modern implant surgery cannot be found in the literature.

The aim of the present study was to evaluate the outcome after perichondrial transplantation to the MCP- and the PIP-joints of the hand, and to compare this historical method with the outcome after modern two-component SR implants. We hypothesized that the medium-term outcome after perichondrial transplantation to finger joints would be at least equal to the results after SR implants. We choose to study the survival rate of the reconstructed joints and did not investigate objective findings and patient reported outcome. Any secondary surgery was defined as endpoint, and the remaining patients were followed to either death or to the end of the study. The MCP- and PIP-joints were investigated separately since they differ considerably in terms of anatomy, surgical technique and outcome [17]. Since the follow-up times differ considerable in the two groups, the secondary aim was to compare the short-term revision rates between the two groups.

## Methods

We designed a comparative retrospective cohort study of patients operated with either perichondrium transplantations or two-component SR implants to the MCP or PIP joints performed at Uppsala University Hospital between 1981 and 2018. The starting point for the time frame, 1981, was chosen in direct continuity to the study cohort from Muder et al. reported earlier this year from our institution [16]. Moreover, since SR implants are relatively new on the market, we wanted a timeframe when both techniques were in use. We have recently reported the long-term outcome after perichondrium transplantation performed before 1981 [16]. None of the cases from before 1981 are included in the present study. Medical records of all patients at the department of Hand Surgery were searched digitally for any of the following codes in the Classification of Surgical Procedures 1997 and the 4:th through 6:th versions of the Classification of Surgical Procedures (valid until 1996): NDM89 (total joint implant in finger), NDB99 (other joint implant in hand or finger joint), NDG29 (other arthroplasty in hand or finger joint), NDN49 (transplantation of cartilage, periosteum, or fascia to wrist or hand), YNA20 (harvest of cartilage for transplantation), 8432 (arthroplasty), 8438 (other reconstructive joint surgery), and 8965 (cartilage transplantation).

**Fig. 1:**
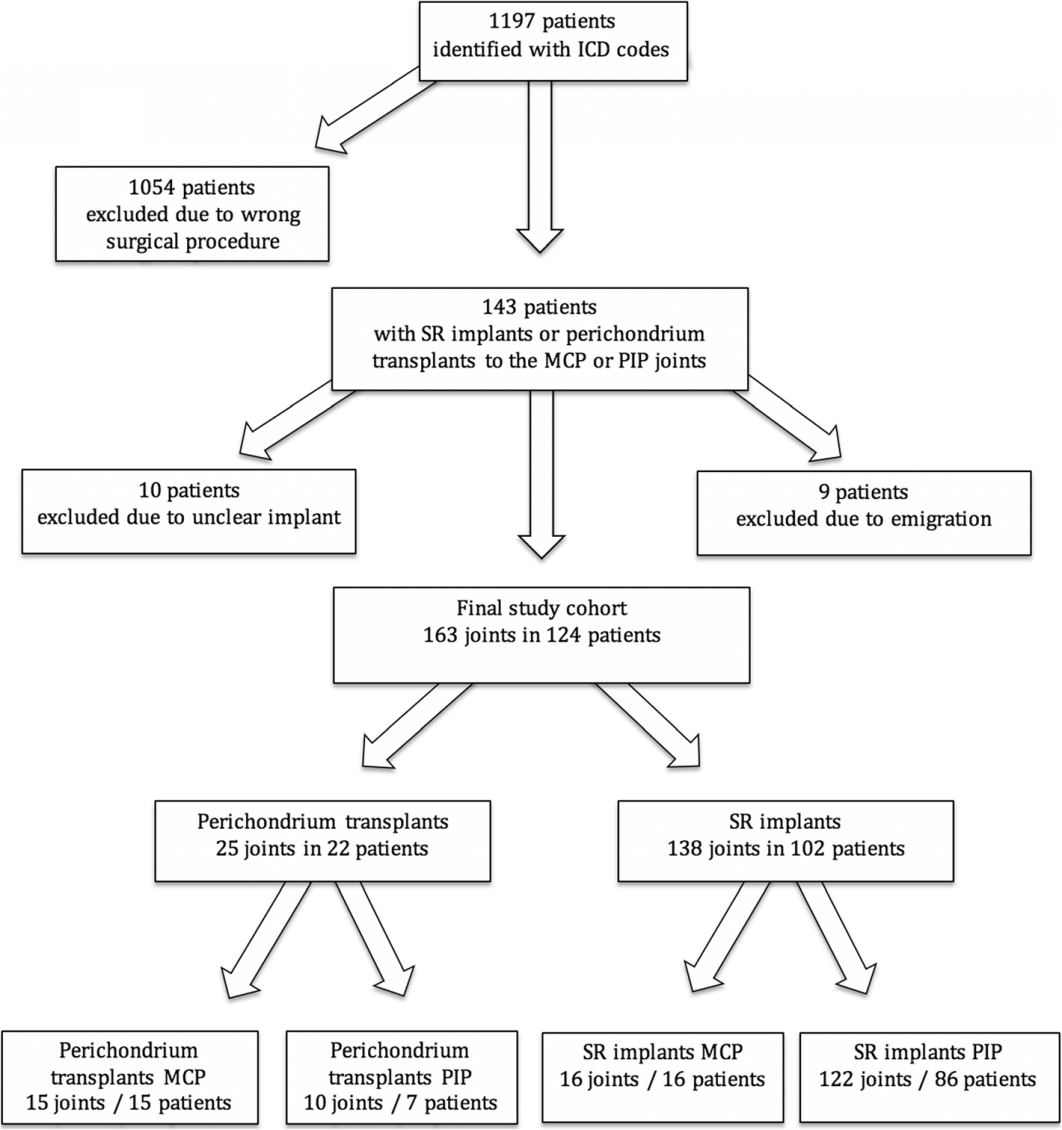
Description of the inclusion of the study population

In all, 1197 patients were identified as potentially eligible along the search criteria given above. Medical charts of all these patients were subsequently searched for operations with either perichondrial transplantation or two-component SR implants to the MCP or PIP joints, and the resulting material was then assessed by reading all operative notes. Nine cases, where the patients had emigrated after the initial surgery and follow-up was impossible, were excluded. Patients where the joint implant could not be clearly identified as a non-constrained two-component implant were excluded (*n* = 10), resulting in 124 patients with 163 operated joints eligible for analysis (Fig. 1). After defining our study cohort, all medical charts were searched for any secondary surgery, including revision, implant extraction, or fusion of the joint. The date of the index surgery, and, if performed, the date of the first revision procedure were registered. Follow-up ended on the day of revision, death, or February 28th 2018, whichever came first.

### Characteristics of the study population

One hundred two patients with two-component SR implants (138 joints) and 22 patients with perichondrium transplantations (25 joints) were included in the study. The groups were further divided into two subgroups describing the MCP (*n* = 31) and PIP (*n* = 132) joints separately. The MCP group consisted of 15 patients (15 joints) with perichondrium transplantations and 16 patients with SR implants (16 joints). The PIP-group consisted of 7 patients (10 joints) with perichondrium transplantations and 86 patients with SR implants (122 joints). In both PIP groups (PIP-transplant and PIP-implant), several patients had surgery to multiple joints. In the PIP-transplant group, one patient had perichondrium transplantation to two PIP joints and one patient had the procedure done to three PIP joints. In the PIP-implant group, 16 patients had implants to two PIP joints, 7 patients had implants to three PIP joints and two patients had implants to 4 PIP joints. None of cases at MCP level (transplant or implant) had surgery to multiple joints.

In the whole study population, the median age at index surgery was 61 years (18–82), and median time to follow-up (revision, death, or February 28th 2018) was 7 years (0–37 SD 8.94). Median time to follow-up was 6 years (0–21) in the SR group and 26 years (1–37) in the perichondrium group.

The surgeries were performed by several different surgeons in both study groups due to the very long study period. The indications for index surgery varied with a predominance of posttraumatic and degenerative osteoarthritis (Table 1). The patients in the perichondrium group were contacted by letter and eleven of them agreed by written informed consent to participate in a clinical follow-up.

**Table 1:** Characteristics of the study population

	Perichondrium (25 joints in 22 patients)		SR (138 joints in 102 patients)	
Joints	MCP	PIP	MCP	PIP
No of joints	15	10	16	122
No of patients	15	7	16	86
Female	1	4	9	74
Male	14	3	7	12
Median age at index surgery 61 (18–82)	44 (18–51)	50 (26–61)	61 (27–78)	64 (24–82)
Posttraumatic osteoarthritis	10	3	2	22
Postinfectious osteoarthritis	3	0	0	0
Degenerative osteoarthritis	2	2	9	71
Rheumatoid osteoarthritis	0	5	5	29

### Surgical procedures

#### Perichondrial transplantation

All perichondrium transplantations were performed under general anesthesia using a dorsal approach to the MCP- and PIP-joints. Briefly, all remnants of the eroded joint surfaces on both sides of the joint were resected down to bleeding subchondral bone with care taken to preserve the shape of joint surfaces. The perichondrium was harvested from the ipsilateral 6th or 7th rib. A skin incision was made in the sub-mammary crease, from the medio-clavicular line and in medial direction. The incision stopped at the medial margin of the crease to avoid unsightly scarring. The rectus abdominis fascia and underlying muscle was divided to expose the donor site. An incision was made along the borders of the cartilaginous part of the donor rib. The perichondrium was lifted at the bone-cartilage rim of the rib and peeled off the underlying cartilage with a blunt dissector all the way to the sternum. The last centimeter of dissection from the medial margin of the skin incision to the lateral margin of the sternum was performed subcutaneously. Care was taken not to harvest any cartilage and not to damage the cambium layer of the perichondrium. The graft was divided in two pieces and trimmed to be slightly larger than the two recipient sites, the two corresponding joint surfaces. The perichondrium was osteo-sutured with 3–0 resorbable monofilament sutures at the joint margins of the recipient site with the cambium layer facing towards the joint space and the outer fibrous layer facing the subchondral bone. A thin silicone membrane (Atos Medical AB, Hörby, Sweden) was temporarily placed in the joint to help mold the transplants and to prevent adherence between the joint surfaces. Minor modifications of the original surgical technique and the postoperative regime were later introduced, in addition to using osteo-sutures, attachment of the graft was reinforced with fibrin glue (TISSEEL, Baxter) [18]. The hand was immobilized in a cast in “the position of safety” [19] for 4–6 weeks, followed by physiotherapy. Extraction of the silicone membrane was usually performed after 6–8 weeks in local anesthesia.

#### SR (surface replacement) implant

Three different types of SR implants were used: *SBI* (Small Bone Innovations Inc.,1380 S. Pennsylvania Avenue, Morrisville, PA 19067 USA), *Avanta* (Avanta Orthopedics, 9369A Carroll Park Drive, San Diego, CA 92121 USA) and *Ascension* (Ascension Orthopedics Inc. 8700 Cameron Rd. # 100, Austin, TX 78754 USA). Eleven MCP implants were cemented, all of them SBI implants. All PIP implants were uncemented. The surgery was performed in either general or regional anesthesia. A dorsal approach with a longitudinal split of the extensor tendon was used for MCP joint replacements. A dorsal approach was also used in majority of the PIP joint replacements (*n* = 91), using the Chamay technique for accessing the joint [17]*.* In a minority of cases (*n* = 31), depending on the surgeons choice, a medio-lateral approach was used, dividing the collateral ligament and leaving the extensor tendon intact [17].

### Statistics

Continuous data were described as medians with ranges or means with standard deviation (SD). Estimation uncertainty was approximated by 95% confidence intervals (CI). Categorical data were summarized in cross-tables and the chi-square test was used to investigate differences between groups. We calculated cumulative unadjusted component survival using the Kaplan–Meier method, with the endpoints extraction or conversion to joint fusion. *P*-values (p) < 0.05 were considered statistically significant. All data were analyzed using the SPSS software version 23 (IBM).

## Results

### MCP group

**Fig. 2:**
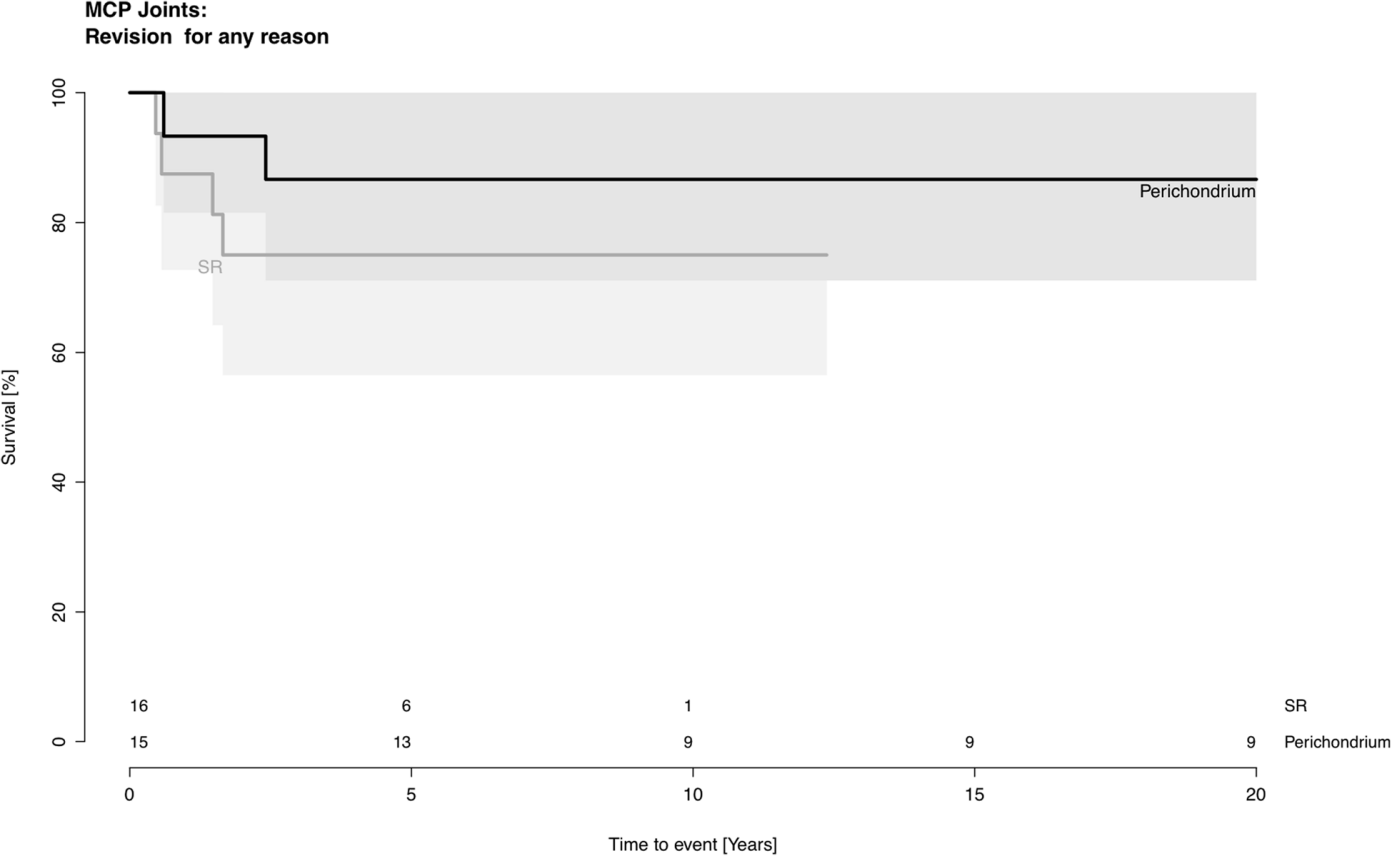
Survival rate at the metacarpophalangeal joint level. Perichondrium transplantation (black) and SR implant (grey)

The revision rate was 25% (4 out of 16 joints) for SR implants and 13% (2 out of 15 joints) for perichondrium transplants (*p* = 0.4). The 10-year joint survival rate was 75% (CI 53.8–96.1) in the SR implant group, and 86.7% (CI 69.4–100.0) in the perichondrium group (*p* = 0.4) (Fig. 2). The median time to revision was 1.0 year (0.5–1.7) in the SR implant group and 1.5 years (0.6–2.4) in the perichondrium group. The median follow-up time was 4.1 years (0–12) in the SR implant group and 21.8 years (1–33) in the perichondrium group. See Table 2 for details. The reasons for revision were mostly pain or joint stiffness in both groups.

**Table 2:** Revisions (*n* = number of joints)

	Implant vs. Perichondrium							
	Implant				Perichondrium			
	Joint				Joint			
	PIP (*n* = 122)		MCP (*n* = 16)		PIP (*n* = 10)		MCP (*n* = 15)	
	No revision (*n* = 93)	Revision (*n* = 29)	No revision (*n* = 12)	Revision (*n* = 4)	No revision (*n* = 8)	Revision (*n* = 2)	No revision (*n* = 13)	Revision (*n* = 2)
Median age at index surgery 61 (18–82)	66 (26–82)	62 (24–77)	63 (27–78)	55 (37–67)	50 (37–67)	55 (53–58)	45 (32–51)	34 (18–50)
Female	82	22	6	3	6	1	1	0
Male	11	7	6	1	2	1	12	2
Post-Traumatic osteoarthritis	14	8	1	1	3	0	8	2
Post-Infectious osteoarthritis	0	0	0	0	0	0	3	0
Degenerative osteoarthritis	59	12	9	0	1	1	2	0
Rheumatoid osteoarthritis	20	9	2	3	4	1	0	0

### PIP Group

**Fig. 3:**
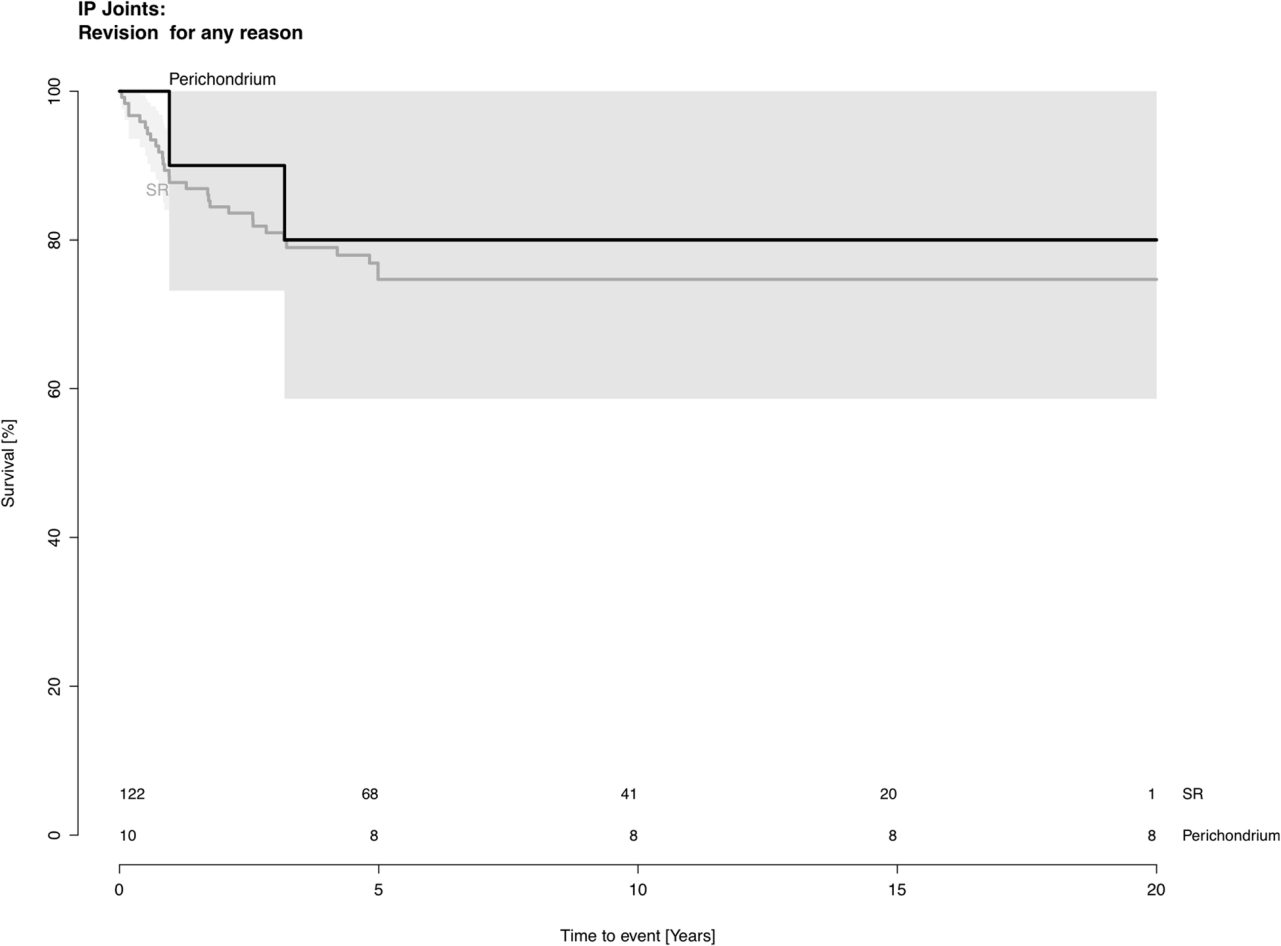
Survival rate at the proximal interphalangeal joint level. Perichondrium transplantation (black) and SR implant (grey)

The revision rate was 24% (29 out of 122 joints) for SR implants and 20% (2 out of 10 joints) for perichondrium transplants (*p* = 0.7). The 10-year joint survival rate was 74.7% (CI 66.6–82.7) in the SR implant group and 80% (CI 55–100.0) in the perichondrium group. (*p* = 0.8) (Fig. 3). The median time to revision was 0.96 years (0.04–5.0) in the SR implant group and 2.07 years (1.0–3.2) in the perichondrium group. The median follow-up time was 6.8 years (0–21) in the SR implant group and 33.8 years (0–37) in the perichondrium group. See Table 2 for details. The reasons for revision consisted either of pain or stiffness in both groups.

### Clinical outcome in the perichondrium group

Eleven of the patients in the perichondrium group (six MCP and five PIP reconstructions) were seen at a clinical follow-up. All measurements and examinations were done by the same observer (DM). Assessment was done with plain radiographs and by measuring range-of-motion (ROM). Manual strength was assessed by a JAMAR hand dynamometer (Patterson Medical Ltd., Nottinghamshire, UK). Pain was assessed with Visual Analog Scale (VAS), (scale: 0 (no pain) to 10 (most severe pain)). Manual ability was assessed with the DASH-score (The disabilities of the arm, shoulder and hand score), (scale: 0 (no disability) to 100 (most severe disability) [19]. The results are displayed in Table 3.

**Table 3:** Clinical outcome in the perichondrium group (11 of 25 patients, 44%)

Perichondrium transplantation	MCP (*n* = 6)	PIP (*n* = 5)
Median age at index surgery (Mean/SD/min-max)	41 years (41/6.6/33–50)	50 years (42/12.8/26–53)
Median follow-up time (Mean/SD/min-max)	23.5 years (19.1/11.8/4–31)	33 years (32.4/1.5/30–34)
Median range-of-motion (Mean/SD/min-max)	70 degrees (66/16.3/40–90)	20 degrees (29/24.6/0–65)
Median pain at rest (Mean/SD/min-max)	0.0 (0/0/0)	0.0 (0/0/0)
Median pain at activity (Mean/SD/min-max)	0.5 (0.6/0.8/0–2)	1.0 (1.4/1.1/0–4)
Median strength JAMAR operated side (Mean/SD/min-max)	38 kg (37.8/9.1/25–52)	10 kg (25.2/16.3/9–52)
Median strength JAMAR non-op side (Mean/SD/min-max)	40 kg (42/8.9/28–53)	10 kg (28.4/26.3/9–62)
Strength in percent op vs non-op side	90%	89%
Median Quick DASH score (Mean/SD/min-max)	8.7 (11.9/10.7/4–33)	8.3 (6.3/3.2/1–8)

## Discussion

The aim of this retrospective cohort study was to compare revision-free joint survival after perichondrium transplantation and two-component SR implants to the MCP and the PIP joints. The major finding in the present study was that the long-term outcome after perichondrium transplantation at least equals the results obtained with modern SR implants. The follow-up time was 20 years longer in the perichondrium group. Patients who were operated with perichondrium transplantation were considerably younger than the patients that were operated with SR implants. At the MCP level, patients with transplants were on average 16 years younger than those with SR implants. At the PIP level the patients with transplants were on average 13 years younger. It might be argued that this age difference would cause bias in favor for the younger group, given that young patients might better cope or adapt to their situation than elderly patients with a SR. However, young individuals usually have higher demands on their hand function in terms of both professional and leisure activities, which in turn increases the mechanical stress on a reconstructed joint. These physical demands tend to decrease with increasing age, and younger patients are exposed to this manual load over a longer period of time. Nevertheless, and despite the age difference, our results show that finger joint reconstruction with perichondrium provide better survival rate than modern two-component SR implants (Figs. 2 and 3).

The two most common causes for SR implant surgery in the MCP group were degenerative osteoarthritis and rheumatoid arthritis. The dominant reasons for perichondrial transplantation at the MCP level were posttraumatic and postinfectious osteoarthritis. The indication for surgery were less diverse at the PIP level, degenerative osteoarthritis and rheumatoid arthritis were most common in the SR implant subgroup, while rheumatoid arthritis was the main reason for joint reconstruction with perichondrium. See Table 1 for details. The diversity in the origin of the joint destruction at the MCP level did not seem to influence the results. The MCP-SR group might be expected to do worse due to impaired periarticular soft tissue in comparison to the MCP-transplant group where posttraumatic and postinfectious osteoarthritis dominated. However, similar outcome figures were found at the PIP level, where the origin of the osteoarthritis was more homogeneous.

Our study indicates lower revision rates after perichondrial transplantation to finger joints than what is previously described. Engkvist et al. reported short-term results (range 3–41 months) of 26 perichondrial transplantations [15]*.* The failure rate for MCP-joints was 28% (*n* = 7) and 42% for PIP-joints (*n* = 7). The surgical indication varied considerable in the study group, e.g., congenital malformations, rheumatoid arthritis, degenerative osteoarthritis, and post-traumatic osteoarthritis that in some cases was associated with significant soft tissue trauma. The operated site also varied considerably; eleven MCP joints, eight PIP joints, four trapezio-metacarpal joints, two metatarso-phalangeal joints of the big toe, and one elbow. The interpretation of the results in such variable cohort becomes difficult and the outcome was, as could be expected, variable. Seradge et al. reported a retrospective study of thirty-six perichondrial transplantations with a minimum follow-up of 3 years [13]. This study group was more homogenous, with sixteen MCP-joints (19% revision) and twenty PIP-joints (30% revision). The results were graded as good if pain was absent, swelling occurred only occasionally, and the range of motion was satisfactory, and such results were found in about half of the MCP and the PIP joints. Muder et al. recently reported clinical outcome in 11 patients 37 years (range 34–41) after perichondrial transplantation to the PIP (*n* = 8) and MCP joint (*n* = 3) [16]. The ROM in the PIP group (n = 8) was on average 41 degrees (range 5–80). In the MCP group (n = 3) the ROM was on average 78 degrees (range 70–90). In the PIP group, grip strength in the operated hand was on average 93% in comparison to the grip strength in the unoperated hand. The same figure in the MCP group was on average 81% in comparison to the unoperated hand. Pain level was low in both groups (VAS 0.6 and 0.7) as well as the DASH-score (8.3 and 2). Perichondrium transplantation has been used to reconstruct other joints like the wrist [12, 20] and the knee [21, 22]. Bouwmeester et al. reported satisfactory medium-term results after perichondrium transplantation to the knee if the chondral defect was isolated, no previous drilling had been done and the patients were < 40 years of age [21]. However, when long-term outcome in this particular cohort (*n* = 14) later was compared with the results after debridement and drilling (*n* = 11) no difference was found [22].

One reason to hesitate about perichondrial transplantation might be the aspect of donor site morbidity. Concerns related to unsightly scarring on the thorax or other complications like bleeding, pneumothorax or nerve damage are not supported by the literature. Moreover, in our experience, the scar is easily and effectively hidden in the sub-mammary crease. Preoperative marking of the donor site incision should be carried out in a relaxed standing position in order to properly identify the medial extension point of the crease. As long as the incision ends at this point the resulting scar does not become unsightly, whereas incisions medial to this point are unnecessary and should be avoided. The last few centimeters of graft harvest towards the sternum is easily done subcutaneously. We have not experienced any hypertrophic scarring or any other donor site morbidity with this technique and could not find any reports in the literature.

Silicone implants are still a reliable method to reconstruct destructed finger joints in the rheumatoid patient, especially at the MCP level. However, in the long term silicone implants have a high fracture rate [23]*,* making their use suboptimal in young individuals and in non-rheumatoid patients.

### Limitations and strengths

**Fig. 4:**
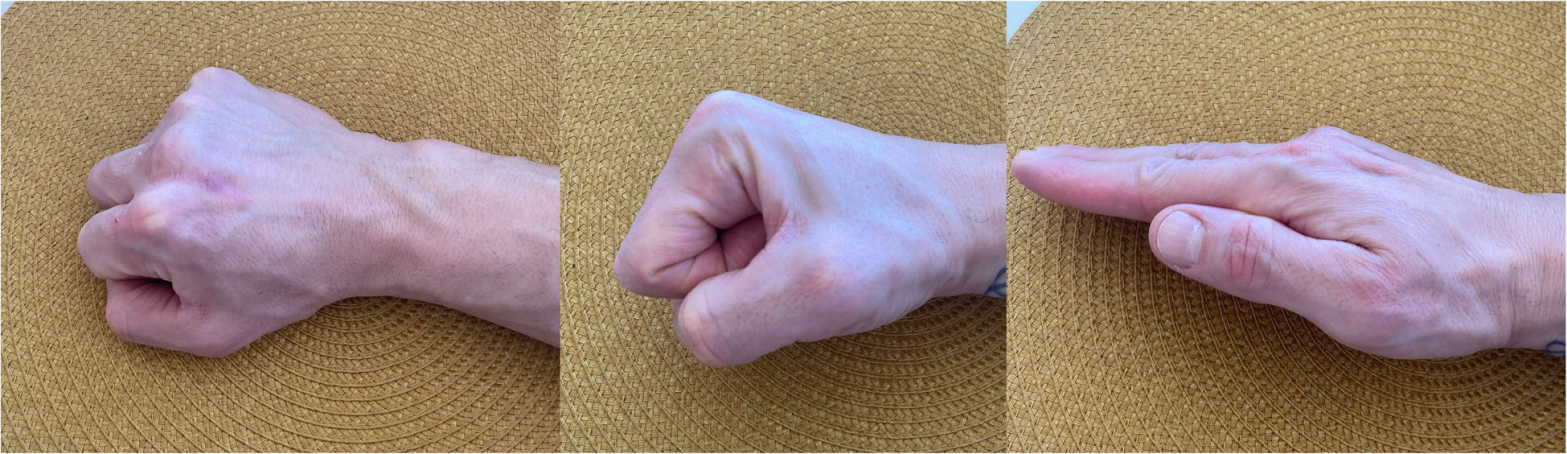
The same patient as in Fig. 6. Present hand function in 2020. Perichondrium transplantation to the MCP joint of the middle-finger was performed 2012


The most important limitation to our study is that it relies on the endpoint revision surgery to describe the results after two very different surgical techniques. It would have been interesting to study objective joint function and patient-reported outcome in this cohort, but given the extensive observation period, we are confronted with large loss to clinical follow-up. The study design is unable to identify unsatisfactory results where the threshold for revision is not met. Such results are probably hidden in both groups. The limited clinical follow-up (11 of 25 patients) in the perichondrium group showed reasonably good, and in some cases excellent, results several decades after the surgery, especially at the MCP level (see Table 3, Fig. 4 and video 1). These results are in accordance to the recently reported long-term results (mean 37 years) after perichondrial transplantation to the PIP and MCP joints [16].

**Additional file 1.** Clinical outcome 8 years after perichondrium transplantation to the MCP joint of the middle-finger. Same patient as in Figs. 4 and 6.

Given the fact that revision surgery is our main outcome, it is important to consider whether such subsequent surgery would have gone unnoticed by us. For the following reasons, we believe this detection bias to be limited: Revision surgery after failed MCP- and PIP-joint implants is only performed at specialized hand surgery units in Sweden, and it is likely that our patients would have returned to the center where the primary surgery was performed. In order to minimize loss to detect revision surgeries, we excluded a number of participants (*n* = 9) from the present study due to their emigration. An additional number of patients (*n* = 10) were excluded since the type of implant could not be unequivocally determined in the operative notes.

Another weakness of our study is the difference in cohort sizes, with relatively few perichondrium transplant patients and joints contrasted by a much higher number of patients and joint with SR implants. In the PIP joint category, the perichondrium group consisted of only 7 patients (10 joints) whilst the SR implant group consisted of 86 patients (122 joints). In the MCP joint category the number of patients in the two subgroups were almost identical but fairly low, 15 and 16 respectively. An alternative would have been to design a propensity-score-matched study, but statistical expertise advised against this due to the scarcity of background information, and the heterogeneity between the two studied groups in terms of age at surgery.

The surgical technique in the patients with SR implants displayed some variation at the PIP level. A small number of surgeries was performed through a lateral approach (*n* = 31) (17)] while the majority of the PIP SR implants were inserted dorsally using the Chamay technique (*n* = 91) [17]*.* However, both techniques are established, and the results fall into the same group and should cause no influence of the comparison with the perichondrium group. Three different types of SR implants were used. They differ in material, the SBI and Avanta implants are manufactured from cobalt-chrome and ultra-high molecular weight polyethylene, whereas the Ascension prosthesis consists of pyrocarbon only. All implants have a non-constrained design with a proximal and a distal component. The perichondrium transplantation technique has been unaltered since 1980’s, when fibrin glue was introduced to reinforce the attachment of the graft [18].

In our hands an implant surgery takes about an hour, while a perichondrium transplantation extends to about 90 min. In addition, a secondary procedure for removal of the silicone membrane is needed in the perichondrium method. This minimally invasive procedure is done in local anesthesia and takes only about 15 min. Economic considerations due to longer operational time and the additional surgery in the perichondrium method should be balanced with the implant costs in the SR group.

Finally, an important weakness of our study is the large difference in the length of follow-up. The median follow-up time in the perichondrium group was 26 years (1–37) while it was 6 years in the SR implant group. We decided to compare these two groups nonetheless and argued that a historical control group is better than none. We subsequently found no statistically or clinically significant differences in early or medium-term survival rates neither in the early revision rate between the two study groups and believe that the observational period is long enough in both groups to enable us to judge whether the medium-term survival after perichondrium transplantation to finger joints at least equals the outcome after SR implants.

### Future studies

Additional studies are needed to clarify the mechanisms by which perichondrial transplant support the formation of a functional joint surface. Modern experimental techniques and approaches have opened new possibilities to study perichondrium and cartilage development and growth [24] and are therefore rapidly evolving the understanding of cartilage development, maintenance, and regeneration [25, 26]. Animal models may help to determine the origin and quality of the resulting joint surface after perichondrial transplantation.

SR implant surgery has increased in popularity during the last decades. The surgery is relatively straight forward and can be done in wide-awake local anesthesia no tourniquet (WALANT) technique [27, 28]*.* In this perspective, it is easy to forget or overlook old techniques such as perichondrium transplantation performed in general anesthesia.

A prospective randomized controlled trial comparing the two methods would provide useful information and help in decision making for each patient but is unlikely to be feasible with such a heterogeneous pathology and patient group. The outcome after SR implant surgery secondary to previous perichondrium transplantation is another interesting topic, especially in comparison to SR implant revision.

**Fig. 5:**
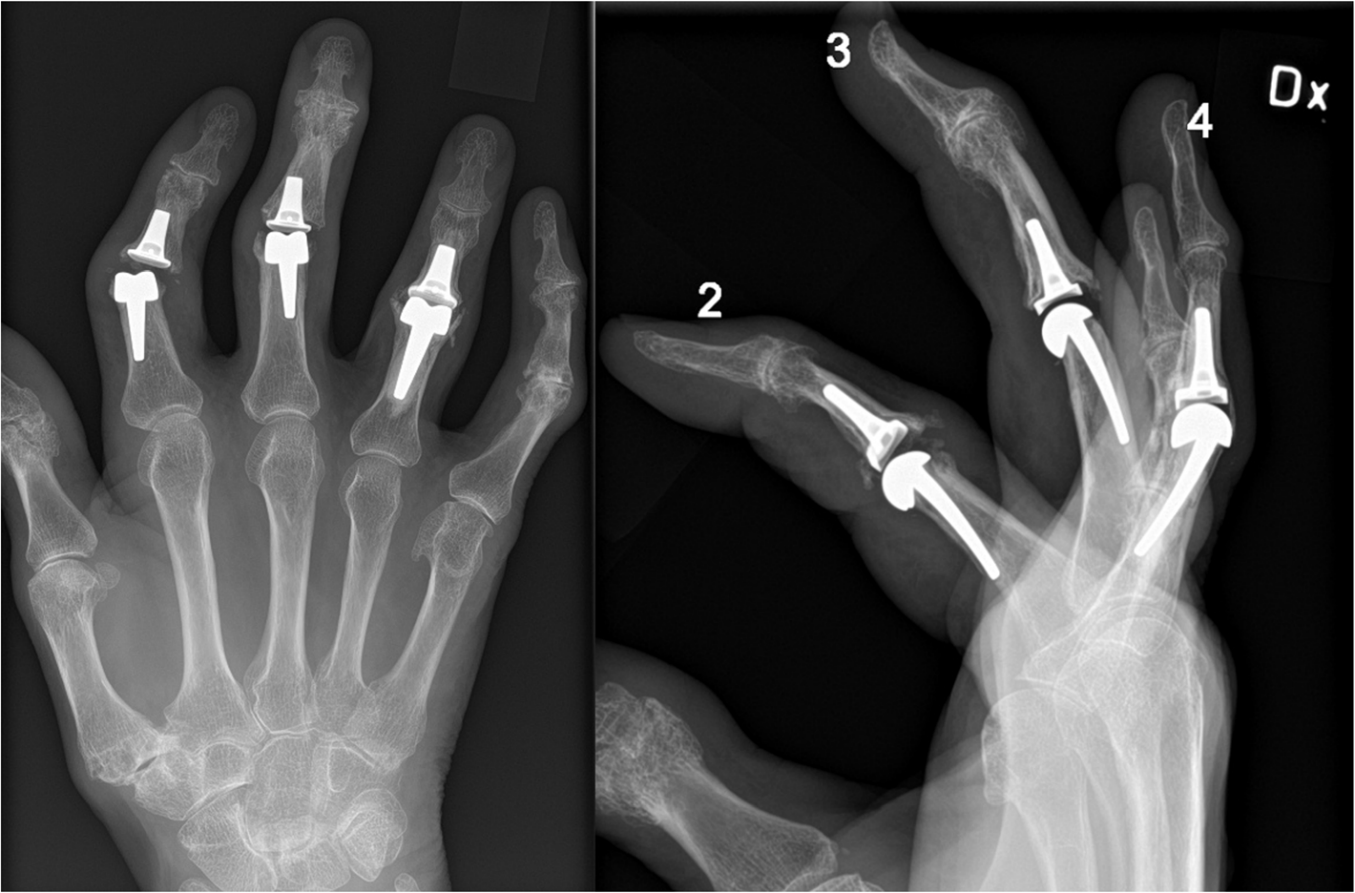
SBI implants at PIP level with different problems. The ring-finger was operated in 2012, and revised in 2015 as the distal component was changed and cemented. Subsequently the joint ended up ankylotic with bony overgrowth. The index and middle-finger were operated in 2016. This radiography from 2017 show signs of subsidence and deviation, especially in the index-finger which later had to be revised and cemented (both components). Only the middle-finger implant has survived without additional surgery

**Fig. 6:**
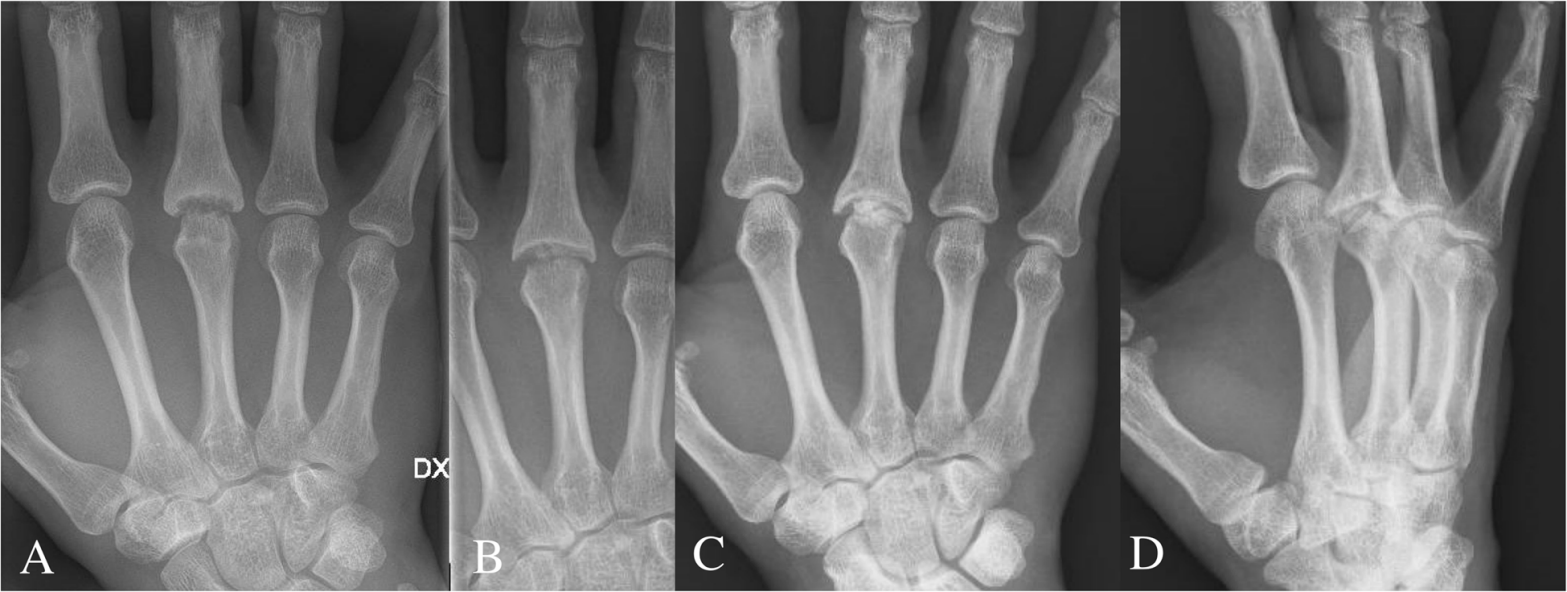
The same patient as in Fig. 4. Fight-bite injury resulting in septic arthritis in the MCP joint of the middle-finger. Reconstruction with perichondrium transplantation was performed in 2012. Preoperative AP view (A). Postoperative follow-up in 2013 (B) and 2016 (C-D). At 4.5 years follow-up the range-of motion was 60 degrees, no pain at activity (VAS 0), no difference in strength op vs. non-op side (JAMAR 52/53 kg) and DASH score 3.3

The major finding in the present study is that the long-term outcome after perichondrium transplantation at least equals the results of modern two-component SR implants. Revision surgery after a failed SR implant is difficult due to the altered anatomy and soft tissue deficiency [29]. Bone graft and/or cement is often needed to compensate for the bone loss (Fig. 5). In contrast, with the perichondrial transplantation technique, the option for later SR implant arthroplasty is preserved since most of the bone and soft tissues around the joint is left intact (Fig. 6). Young individuals operated today with perichondrium transplantation probably have better implant options in the future if they end up needing a revision later in life. They have become older and further improvement in implant survival rate could be expected.

## Conclusion

In conclusion, reconstruction of osteoarthritic finger joints with rib perichondrium is a technique worth considering. The relative preservation of the bone stock and soft tissue surrounding the joint is attractive, especially in young individuals. It may provide an alternative to conventional arthroplasty, and later conversion to SR implants is still possible.

## Data Availability

The data set supporting the conclusion of this article is available on request to the corresponding author.

## Abbreviations

SR: Surface replacement
MCP: Metacarpophalangeal
PIP: Proximal interphalangeal
CI: Confidence interval
N: Number
Fig: Figure
SD: Standard deviation
IBM: International business machines corporation
ROM: Range of motion
VAS: Visual analogue scale
DASH: Disabilities of the arm, shoulder and hand
WALANT: Wide-awake local anesthesia no tourniquet
